# A Clinical Lecture on Spasmodic Dysmenorrhœa

**Published:** 1902-03-01

**Authors:** D. C. Rayner

**Affiliations:** Lecturer on Practical Midwifery, University College, Bristol


### Hospital Clinics and Medical Progress.

### A CLINICAL LECTURE ON SPASMODIC DYSMENORRHŒA.

By D. C. Rayner, F.R.C.S. (Eng). Lecturer on Practical Midwifery, University College, Bristol.

Of all the different varieties of dysmenorrhœa, the most interesting and perhaps the most important is that known as "spasmodic" dysmenorrhœa. It is most interesting on account of the uncertainty that exists as regards its pathology; and most important on account of its frequency and the severity of the pain with which it is accompanied. This disease is called "spasmodic" because it is believed that the uterine contractions which accompany menstruation instead of being as in health slow and gradual, are "spasmodic"; that is, violent, sudden, and painful.

We will first of all consider some of the theories that have been brought forward as to its cause; then the symptoms of a typical case; and lastly, the different methods of treatment that have been undertaken for its relief.

First of all with regard to the causes of spasmodic dysmenorrhœa we must consider briefly some of the different theories that have been brought forward. (1) The obstructive theory: It was at one time thought that the pain was due to a certain amount of stenosis of the cervix, which interfered with the discharge of blood from the uterus. There are many objections to this theory. In many of the most severe cases there is no stenosis, and some women with most marked anteflexion and a pin-hole os menstruate with no abnormal discomfort. Then again the pain usually precedes the flow, diminishing as it appears, and ceasing at the end of the first or second day. This proves that the dysmenorrhœa is not due to obstruction, for in that case the more there was to pass, the worse would be the pain, whereas women constantly declare that they have most pain when they have the least flow. During the progress of the pain also it is easy to prove the absence of obstruction; for at the very acme of the pain, a few hours before the flow appears, a bougie No. 7, 8, or 9, will pass easily, though perhaps painfully, along the whole length of the uterine canal. (2) The theory that it is due to some alteration in the polarity of the uterus: This means that there is an antagonism between the body and cervix uteri. It is supposed that normally when the body of the uterus contracts the cervix dilates, but that in spasmodic dysmenorrhœa the absence of physiological relaxation of the cervix prevents the normal, regular, painless contraction of the body. This pathology rests upon inference only; we do not know from direct observation that the uterine canal is smaller during the pain of spasmodic dysmenorrhœa than is the canal of a patient who is menstruating painlessly. (3) That the pain is due to an abnormal sensitiveness of the os internum: In a healthy woman the internal os uteri and the whole interior of the uterus are sensitive—that is to say, the touching of them by a probe is disagreeable. In a woman suffering from spasmodic dysmenorrhœa the pain on touching the internal os is intense, and will often bring on severe spasms which last for some time after the removal of the instrument. (4) Defective development of the uterus: In many of these cases the uterus is found to be in a more or less infantile condition. An undeveloped organ performs its functions badly, and the uterus is no exception to the rule. Sir John Williams has specially emphasised the connection that often exists between imperfect development of the uterus and dysmenorrhœa. Symptoms of spasmodic dysmenorrhœa: The history of a typical case is usually as follows:—The patient is seized with violent paroxysmal pain in the centre of the lower abdomen, generally some hours before the commencement of the flow. The pain is bilateral and referred like that of labour to the lower part of the abdomen and back, each paroxysm being described as lasting from about one to ten minutes. The pain is more severe than any other kind of menstrual pain, and is sometimes accompanied with vomiting and with severe headache somewhat resembling migraine. The duration of the attack is variable, in some the severe pain only lasts a few hours, in others as long as 24. During the height of the pain, the flow is usually scanty, and when the blood begins to escape freely the pain is often relieved, and indeed may be absent during the remainder of the menstrual period. The period is usually followed by several days of great physical weakness and debility. Unless relieved by pregnancy, or by proper treatment, the condition will persist through the menstrual life of the patient, and the suffering tends to increase with time, and later to become complicated by endometritis, salpingitis, and ovaritis. Treatment: In a typical case of spasmodic dysmenorrhœa but little relief can be obtained from drugs. Of course the general health of the patient should be attended to, and in the case of an unmarried girl no local treatment should be undertaken unless the pain is severe and drugs have been given a fair trial. The great number and variety of drugs that have been recommended is in itself a sufficient proof that they are usually inefficient. Amongst those that have been used for the relief of the severe pain are, antipyrine, phenacetin, exalgine, nitro-glycerine, viburnum prunifolium, etc. Of these the ones I have found to give most relief are antipyrine, and phenacetin, 10–15 grains of the former, combined with half a drachm of sp. chloroformi or sp. ammon. aromat., and repeated if necessary. If antipyrine fails phenacetine in 10 grain doses may be tried. There is one drug, however, which we should be very reluctant to prescribe, and that is opium. The disease is a chronic one; it is likely to recur every month for a considerable time, and you are in very great danger of teaching your patient the opium habit, which is a very much greater evil than the one you hope to cure. Immense harm is often done by giving a prescription of opium for dysmenorrhœa. If the pain is unrelieved by drugs local treatment must be undertaken, and this consists in dilating the cervical canal. This operation if undertaken with due care, and with strict antiseptic precautions, is practically without risk; in the absence of these, however, it may be followed by very serious consequences. Division of the external and internal os by means of scissors or a metrotome, are now abandoned, as they possess no advantage over dilatation and are attended with considerable risk. There are three chief methods of dilating the cervix, (1) by the successive passage of bougies of gradually increasing size, (2) by the introduction of a two-, three-, or four-bladed dilator, the blades of which are separated by a screw, (3) by means of tents. Of these three methods I prefer the first. The operation : The patient having been prepared by previous purgation, the vagina having been douched and strict antiseptic precautions taken, she is anæsthetised and placed in the lithotomy position. The vagina is then again douched with a 1-2000 sublimate solution, the anterior lip of the cervix is now seized with a pair of strong vulsellum forceps, drawn downwards, and held steady. Bougies of gradually increasing size are then passed, and dilatation carried to such a degree as to thoroughly stretch the canal without tearing it. If there is any endometritis present the uterus should be curetted at the same time. The patient must be kept in bed for a week and antiseptic douches used daily. Rapid dilatation by means of two- or three-bladed dilators is preferred by some, and if due care is exercised and dilatation carried out slowly there is very little risk of tearing the cervix, which is one of the chief objections to their use. Dilatation by means of tents possesses no advantages over graduated bougies ; if they are used laminaria tents are the best. The effect of this simple operation in most cases of true spasmodic dysmenorrhœa is often very remarkable ; in others, although the pain is not completely cured, it is greatly ameliorated, and failure is generally due to the case not being one of the true spasmodic variety.

